# The Role of Mental Health and Sustainable Learning Behavior of Students in Education Sector Influences Sustainable Environment

**DOI:** 10.3389/fpsyg.2022.822751

**Published:** 2022-02-08

**Authors:** Zhaoliang Gu, Pu Li, Aiai Zhang, Xiaoqiang Xu, Fengmiao Gu

**Affiliations:** ^1^School of Marxism, Chengdu University of Technology, Chengdu, China; ^2^College of Management Science, Chengdu University of Technology, Chengdu, China; ^3^Logistics Service Group, Chengdu University of Technology, Chengdu, China

**Keywords:** mental health, sustainable environment, e-learning, psychological motivation, learning management, wellbeings

## Abstract

Mental health has been declared as the essential component of overall human wellbeing. However, there has been a very steep rate of depression and anxiety in students that exhibit their social and personal burdens. It has been widely accepted that the wellbeing and mental health of individuals are a mix of psychological, genetic, social, lifestyle factors, and environmental exposure. Due to the pandemic, the shift from traditional classroom learning to e-learning has also disturbed the mental health of students, which consequently affects environmental stability. The current study has also measured the effect on the mental health of e-learning behaviors (psychological motivation, peer collaboration, cognitive problem-solving, interaction with the instructor, community support, and learning management). The population of the study was the undergraduate students enrolled in the colleges of China, and they were chosen *via* convenient sampling. The findings of the study show that mental health has a significant positive effect on the e-learning behavior of the students and consequently affects environmental sustainability. Educational institutions are improving their e-learning programs by understanding the preferences and challenges of students regarding online learning. Educational institutions should revise their policies on online education and teaching methodologies. Furthermore, the current study has taken undergraduate students as the sample. In future studies, these relationships can be checked in higher education as well.

## Introduction

According to the United Nations Educational, Scientific, and Cultural Organization, the progress of a country depends on the literary abilities of its residents (Raji and Zualkernan, 2016). Since countries that are better equipped for literacy can help better in the advancement and development of their societies, therefore, these countries make heavy investments in learning, vocational, and education programs (Raji and Zualkernan, 2016). Along with investments in educational programs, the mental health of students has also been found to be very critical to a positive contribution to society and the environment (Lister et al., 2021). Just like conventional learning methods, the current mode of online learning also has a certain role to play in the attainment of a sustainable environment, considering the mental health of students due to this abrupt shift in the learning mode. Therefore, the current study explores the underlying effect of the mental health of students on their electronic learning (e-learning) and their role in achieving a mentally stable and sustainable environment.

Considering nature’s positive impact on mental health, addressing environmental problems can give winning solutions to the increased number of mental health problems and economic regression that arose during the current pandemic. The natural environment is considered one of the key concerns of mental health. This pandemic urges policy makers to devise such policies that address the mental health of individuals to promote a better and healthier future (Lister et al., 2021). Because the environment can affect the mental wellbeing of individuals either positively or negatively and *vice versa*, the effects of changing paradigms, including job insecurity, economic uncertainty, volatile and extreme weather patterns, and displacements, are the triggering factors in mental health deterioration.

The realization of the fact that health and the environment are related to each other has grown stronger over the years (Lister et al., 2021). Human beings are an integral part of the ecosystem of the earth, which is defined as the interaction of humans with their social, biological, physical, and chemical environment. Contrasting the other factors of the ecosystem, humans have evolved over the past few thousand years not only physically but also socially and culturally, which has made them stronger constituents able to control their environments rather than being completely bent by them. This evolution has been both beneficial and harmful to the environment itself (Lister et al., 2021). Biological, physical, and chemical hazards to the environment are associated with the advancement of human technology, especially in the fields of construction and production (Qin et al., 2021; Yingfei et al., 2021). However, these damages can be controlled even at present through learning and improving mental health, thus working in the direction that leads to a win-win situation for the environment and learning simultaneously (Nawaz et al., 2021).

Scientific development and technology have been found to be an important reason for advancement in the field of health on a large scale in the whole world. As the advancement in technology has paced, the media of education have also been updated side by side with online learning, which has affected the mental health and academic performance of students (Deshpande and Mhatre, 2021). The current pandemic has been one of the reasons for these broad changes in the dynamic of students’ everyday lives because lockdowns all over the world have shifted conventional classroom learning to online learning (Patricia, 2020; Deshpande and Mhatre, 2021). This transition of education from conventional to online learning was haphazard due to the stress that students faced in this new mode of learning and difficulties in understanding and completing their home tasks. Apart from this, the mental health of students was immensely affected in the form of depression, anxiety, stress, frustration, and uncertainty due to the pandemic (Hao et al., 2020; Nawaz et al., 2020a; Huo et al., 2021). However, not only is learning for the sake of knowledge, but learning that contributes to the sustainability of the educational environment and learning itself is very important (Raji and Zualkernan, 2016).

The study is primarily motivated by the goal theory: a motivation for achieving a sustainable environment by instilling the intrinsic motivation for e-learning in the e-learners of the pandemic era and the theory of sustainability that explains the achievement of optimum utilization of resources without compromising the availability of future resources (Ciocoiu, 2011). Therefore, in this unprecedented period when everything is changing at a fast pace, especially the health conditions due to pandemics when studies have also turned to electronic learning, it is important to keep the vulnerability of the mental health of people less vulnerable to such changes and their role in achieving a sustainable environment. In this regard for achieving a sustainable environment, considering the higher education and e-learning post-pandemic, there lies the understanding at three stages: (a) first of all understanding what is environmental sustainability in this perspective (ontology), this means the issue of pandemic prevails globally and the responsibility lies with the whole world working collectively for its measures (Nawaz et al., 2020b); (b) making people aware of this sustainable environment through e-learning (epistemology), i.e., in what possible ways mental health of the e-learners can affect their e-learning and the consequent effect on the attainment of sustainable environment, and (c) supporting this transformation for the sustainable environment since the theory of sustainable environment connects the knowledge and social actions of the e-learners (ethical consideration). Hence, the current study aims to understand the role of mental health in the e-learning behaviors of students and how much it helps in achieving a sustainable environment. In this regard, the main objectives of the study that address the research questions are described in the following:

(1) To understand the role of mental health in the online learning behaviors of students. This means that mental health lies at the preliminary stage when it comes to social interaction and not the other way round. This study particularly considers the context in which mental health is the predictor of e-learning behaviors and a sustainable environment.
(2) To identify the effects of e-learning behavior in achieving the sustainable environment.
(3) To know how much e-learning mediates the relationship between mental health and a sustainable environment? E-learning behavior has been the composite of certain sub-variables in the present study, which affect the mental states of the e-learner, which are major contributors to the sustainable environment.

The current research paper has been organized into eight sections in total. The first section introduces the variables and explains the significance and rigor of the study, along with its objectives. In the second section, the literature is reviewed. The third section addresses the conceptual framework and the theories that formed the basis of it. The fourth section explains the research methods used in the study. In the fifth, section, the results are discussed in accordance with the previous pieces of research. In the preceding sections, the conclusion, practical implications, and limitations have been reported for the study.

## Review of Literature

### Mental Health

Mental health disorders are on the rise in western countries (Ohrnberger et al., 2017). The outbreak in the world has changed all activities, not only social, educational, cultural, but also individual lives. This has been the biggest shock to health in so many years lately (Aucejo et al., 2020). Similarly, students have also been the most affected segment of the population due to the shifts in educational paradigms. School routines have been found to be very important for coping with the mental stress of students. The students had been facing fears of losing their academic years, becoming seriously sick, or lacking resources to complete their studies. Furthermore, the transition from classroom learning to online learning in colleges and universities has immensely affected educational plans, academic performance, the labor market, and expectations for future employment opportunities (Aucejo et al., 2020).

Students have been under the shock of a pandemic related to its outcomes, such as anxiety, loneliness, social distancing, and the physical health of being contacted with the virus. The mental health of individuals is adversely affected by any negative or unexpected events. However, how this mental health can affect the education and environment of the university is still unknown but is predicted to be substantial (Gallego et al., 2020; Odriozola-González et al., 2020). Emotional disorders are mostly seen in college students, and the presence of such disorders in this time of pandemic is supposed to produce adverse effects on the minds of the students (Saha et al., 2021). The pandemic has not only affected the health, but it has developed the concerns of people regarding their education, mental health, stress level, anxiety, relationships with others, and overall wellbeing of the families.

The developmental systems theory states that there have been certain environmental factors that have mediating effects on the mental health of individuals after they have gone through some trauma or undesired circumstances (Ye et al., 2020). Social support, effective coping strategies, and resilience explain the underlying reasons for the mental trauma and health of the individuals. Perceived stress and mental health have been associated lately with the mediation of social support as trauma in life makes people more traumatized at the expense of their mental health due to a lack of social support-seeking behaviors (Nawaz et al., 2020a).

Similarly, students living in combined family systems tend to have more social support and are found to have more stable mental health than those who are left alone in nuclear families (Noor et al., 2014). Therefore, social support and peer collaboration have been found to be strongly associated with the mental health of students. Similarly, mentally stable individuals tend to deal with their life matters more intelligently and emotionally than those who are unstable in these respects. Strong effects of mental health have been found in the physical health of individuals. However, they have not been precisely mentioned as to what could be the consequences in affecting the real-life experiences (Ohrnberger et al., 2017). Therefore, understanding the role of mental health in the expectation of deviance from normal behavior is very important if these concerns need to be addressed. Therefore, the current study attempts to check the effects of mental health of the students on their online learning behaviors to explore what helps and what does not help them in achieving their desired goals in the educational stream and hence an overall sustainable environment. The hypotheses have been devised with the subsequent mediating and consequent variables of online learning and sustainable environment that are mentioned in the following sections.

### Sustainable Development

Generally, according to Biasutti and Frate (2017), the environment is about raising awareness about the resources, the tenderness of the physical surroundings, and the vulnerability of the healthy environment. Maintaining a good and healthy environment around our activities is very important. The environment includes natural resources, rural development, change in climate, sustainable urbanization, mitigation, and disaster management. The decisions and activities of the humans living in a locality make an intense effect on their environment, not only by the common man but also by the social, political, and economic developments made at a mass level (Biasutti and Frate, 2017).

Sustainability has been associated with knowledge in several ways as the actions associated with sustainable development. It is crucial to raise public awareness about environmental issues by increasing the number of learning courses at universities regarding advanced communication and information technologies (Badea et al., 2020; Nie et al., 2020). Execution of such strategies requires the organizations to reach flexibility, develop new competencies, and engage in competitions with other educational institutes. Competency in sustainability is associated with skills, acquired knowledge, and relationships that enable desired task performance and sustainable problem-solving (Sivapalan et al., 2016; Nie et al., 2020; Syakur and Sabat, 2020). Hence, in the future, advanced technologies should be fully integrated into the learning models to reap the full fruits of sustainable e-learning developments (Nie et al., 2020). Sustainability in learning is about gaining additional knowledge based on social learning and empowerment. The learning models containing foresighted thinking, self and other motivations, planning, and implementation are supposed to enable the students to move toward more sustainable social models.

### E-Learning and Sustainable Environment

E-learning is closely linked to sustainability and flexibility in planning, financing the courses, outreach, reporting, and assessment (Lozano et al., 2013). According to Badea et al. (2020), sustainable development goals are employed in higher education, which can be used in shaping sustainable behaviors by exercising education for sustainable development. They also found a relationship between the students’ behaviors and educational sustainability. The pervasiveness of the sustainable development goals requires social and environmental sustainability along with economic sustainability. Education for sustainability has been defined as the teaching and learning approach that is based on the principles and standards to train people to plan, cope, and find the solutions to problems without causing any threat to the environment. Hence, it includes the involvement of those educational activities that do not cause harm to the environment but rather help in sustaining it.

At the individual level, sustainability demands responsible behaviors from the individuals that help in maintaining the sustainability of the environment (Badea et al., 2020). It has been found in so many studies that the irreversible damage to the environment is mostly caused by the production of an industry’s extensive use of available resources that damage the planet. Limiting the behaviors that are unsustainable for the environment can help in decreasing the deterioration of the surrounding at the mass level; hence, to shape the behaviors of people education is necessary (Shephard, 2015; Caniglia et al., 2018; Calvo et al., 2020; Lister et al., 2021). Educating the younger generation in such a way creates the attitudes, responsibilities, skills, and understanding of the concept of environmental stability in their minds as children (Mahat et al., 2017; Badea et al., 2020). It has also been argued that as adolescents in their secondary schools, it is easier to implant the values since they are more receptive and can easily develop sustainable behavior and also induce this in others (Calvo et al., 2020; Nie et al., 2020). Students are the future entrepreneurs and policymakers who will be in charge of environmental management by making a significant difference in terms of acquiring and preserving good habits for a sustainable lifestyle (Badea et al., 2020). Therefore, the students engaged in e-learning and multidisciplinary interaction tend to develop a sense of environmental preservation and sustainability in the future. To find this, the following hypotheses have been devised by breaking down the e-learning factors to find their role in attaining environmental sustainability.

### Sustainable E-Learning Behavior

The ability to read and write is basic to individuals. The illiteracy of nations is connected to poor educational initiatives and the lack of adoption of the new technologies emerging globally (Raji and Zualkernan, 2016). Learning interventions are found to be necessary to make the residents of the country aware of state-of-the-art techniques. Such learning interventions could include anything used widely in the world, like mobile learning (using mobile phone applications for learning), electronic learning (e-learning, using any online gadget to access the new technology either in the form of a laptop, desktop, iPad, or Thinkpad, etc.) (Knowles, 2001; Asarta and Schmidt, 2020; Bao, 2020; Hasan and Bao, 2020). However, it is necessary to choose and use the most suitable and adequate technological intervention for learning.

Learning technologies for e-learning encompass an extensive range of educational programs that adhere to information, communication, and technologies that support or can be used to hold up the learning, teaching, and assessment of these activities (Raji and Zualkernan, 2016; Lock et al., 2021; Thandevaraj et al., 2021; Zhai et al., 2021). E-learning can be defined as a process of learning and teaching following a proper model of distance learning with flexible education focused on learning behaviors (Nie et al., 2020). It usually takes place in a virtual learning environment, in which multiple directions and multi-facet discussions are possible. Some of the educational institutes make their degree programs online, including the asynchronous classes, by recording the lectures and making them available online according to the schedule (Patricia, 2020). There have been many online learning modes used by the learner and/or trainer depending on their needs. Such online intervening technologies have led to different online learning programs, such as educational TV channels, educational radio programs, mobile phone applications, online resource learning, massive open online courses (MOOC), and remote online tuition.

The physical classroom learning of the students has shown the rich interaction between the students and the teachers and the students with the students (Zhou and Zhang, 2021). However, due to the spread of the pandemic and social distancing in the classrooms, there have been many cases of anxiety and depression among the students, higher dropout rates, and less engagement of students due to remote distance among students and teachers (Lee et al., 2019). Although e-learning has been the safest mode of learning considering the pandemic, it has caused a feeling of loneliness and confinement among the students by limiting the earlier student-to-student and student-to-teacher interactions. One of the main objectives of the present study is to measure the effects of the mental health of e-learners on sustainable e-learning behaviors. Sustainable e-learning behavior has been taken from previous studies that have successfully measured it with the presently used variable. It has been split into six sub-variables, namely, psychological motivation, peer collaboration, cognitive problem-solving, interaction with the instructor, community support, and learning management (Lee et al., 2019).

Odriozola-González et al. (2020) and Zhou and Zhang (2021) applied some tests to the results of the online learning and conventional classroom teaching classes and found that the students had shown better performance in their online learning classes as compared to the previous year of face-to-face learning. E-learning has been found to have many positive outcomes such as higher-order cognitive skills and higher learning achievements because the students engaged in online learning can access the learning material anywhere and anytime (Lee et al., 2019). Student engagement has been explained by concluding the mix of behavioral and emotional factors. The behavioral factors of students are characterized by the attitude and involvement of the students in lectures, asking questions, and meeting the deadlines for the assignments. However, the emotional factor includes the sentiments of the students toward learning and the feeling of affiliation with their learning community (Lee et al., 2019). There have been six major factors identified by Lee et al. (2019) that explain the sustainability of e-learning behaviors. The current study uses those six factors to identify the sustainable e-learning of students. These include psychological motivation, peer collaboration, cognitive problem-solving, interaction with the instructor, community support, and learning management.

#### Psychological Motivation

The learning environment has been found to strongly influence the motivations of the students (King and Bunce, 2020). The impact of teaching methodologies on the student’s mental health is found to consequently affect the development of positive or negative motivations among the students. When the students have an active motivation to learn something, they prove themselves as active learners. The mental health of the students has been found to have detrimental effects on the students’ motivations and teaching styles (McIntyre et al., 2018; Serafini et al., 2020; Saha et al., 2021; Usher et al., 2021). Any change in the physical features of the environment in which students operate actively affects the motivation and cognition of the students (Schunk and DiBenedetto, 2020). At all levels of learning and education in universities and colleges, the social and personal lives of the students have changed drastically due to the pandemic, which has led to a shift in their psychological and behavioral aspects (Kazak, 2020). Usher et al. (2021) found that the change in the mode of learning regarding the shift from conventional learning to remote learning has affected the mental health of students who have turned to behavioral and psychological motivations for change. It has also been found that students have self-reported, in an open-ended survey, that anxiety caused by the pandemic has changed their behaviors and psychological motivations (Usher et al., 2021). Hence, it is important to understand how these sudden shifts in the learning modes of students change the psychological motivations and academic experiences of the students, considering their mental health and self-regulation. Therefore, the following hypotheses have been devised.

*H* _1_
*: Mental health has an effect on the psychological motivation of the students.*
*H* _2_
*: Psychological motivation has an effect on a sustainable environment.*
*H* _3_
*: Psychological motivation mediates the relationship between mental health and a sustainable environment.*

#### Peer Collaboration

Peer collaboration means the involvement of learners in mutual and combined activities in which they discuss and transfer their knowledge in order to collaboratively solve the problems (Lee et al., 2019). When the students working together are in a sound state of mind, they tend to help each other in their learning activities and enhance their output when working in teams (Tsai and Yin, 2019). Peer collaboration has been found to be a very effective method of teaching in classrooms, considering the small group of individuals or working in pairs by indulging them in cooperative activities to reach a higher level of satisfaction (Nunkoo and Ramkissoon, 2011; Tsai and Yin, 2019).

When the students collaborate with each other, they tend to brainstorm together, discovering each other’s new ideas, and it can help the low achievers by making teams with the high achievers. These peer collaborative activities and encouragement have been found to be effective as positive reinforcement for students, which is possible only when the counterpart is in good mental health. Peers have been found to have the potential to enable the learning contexts that can profoundly impact the students’ development (Wentzel and Watkins, 2002; Baig and Waheed, 2016). The collaboration among peers promotes collaboration in the academic engagements and supporting structures for the improvement of quality of education, acquiring the skills, knowledge, and values for redefining the curriculum that is necessary for achieving sustainability through public awareness for better understanding and reaching the sustainable environment (Nunkoo and Ramkissoon, 2011; Nie et al., 2020). For this reason, mental health has been taken as the predictor of peer collaboration, not the other way round, because sound mental health makes individuals indulge in healthy social activities with their peers. On the basis of the literature, the following hypotheses have been formulated.

*H* _4_
*: Mental health of students has an effect on peer collaboration.*
*H* _5_
*: Peer collaboration has an effect on a sustainable environment.*
*H* _6_
*: Peer collaboration mediates the relationship between mental health and a sustainable environment.*

#### Cognitive Problem-Solving

The use of technology has revolutionized human activities in so many ways. Literature has identified so many activities but has highlighted the three main activities, particularly in the education sector, such as improved efficiency, complex problem-solving, and analyzing the variables in the best way (Waheed and Baig, 2017; Molnár and Csapó, 2018). Complex problem-solving is related to higher-order cognitive processes. There have been developed certain problem-solving strategies that can be employed by the students in complex situations to get the optimum solutions, more intensely the win-win solutions causing lesser damage (Süß and Kretzschmar, 2018; Lee et al., 2019). Kim et al. (2018) have found that the cognitive skills should be embedded in the individuals in their universities to develop them as behavior and identify the new opportunities right in time. They have found a significant relationship between the cognitive skills of the students in innovative behavior, problem-solving, and the ability to perceive opportunities with better options.

Problem-solving skills have been found to be a critical factor in organizations and the success of professional careers (Süß and Kretzschmar, 2018). Previous literature has defined cognitive problem-solving as a sophisticated and volatile technology-based technique to get the optimum and best possible solution. Furthermore, it has also been argued that individual problem-solving techniques are important in improving problem-solving abilities (Kim et al., 2018). Some problems require prior knowledge along with intellectual abilities to solve which is the reason for problem-solving in the best possible way. There have been past studies that have found that cognitive abilities have been applied in the teaching methods but not in the entrepreneurial studies to find innovative ideas (Kim et al., 2018). Furthermore, it has been stressed that the behavioral curriculum should be added to embed the cognitive skills as behavior in the students, hence making the inclusion of cognitive skills polishing an important factor in sustainable e-learning behavior. When the problems are solved in optimal ways, e-learning encourages the students to solve their problems in a way that maximizes the pros for their colleagues and the outer environment as well. Hence, the following hypotheses regarding the role of cognitive problem-solving in environmental sustainability have been mentioned.

*H* _7_
*: Mental health of students has an effect on cognitive problem-solving.*
*H* _8_
*: Cognitive problem-solving has an effect on a sustainable environment.*
*H* _9_
*: Cognitive problem-solving mediates the relationship between mental health and a sustainable environment.*

#### Interaction With the Instructor

When students are involved in online learning, they should be satisfied with their modes of learning and the content offered. Furthermore, in order to make a community bond with the students, it is important for the instructors to focus on the activities that enhance learner and instructor interaction easily and frequently. Interaction has been defined as an event that involves the communication of two objects or actions (Shackelford and Maxwell, 2012). In an interaction with the students, the instructor tries to develop the course content in a way that motivates the students to give attention and helps them in their learning processes. It is necessary for the instructors to make the environment of the community one that helps in developing the community (Coşkun and Kara, 2020). In addition, it is also in the hands of the instructor how the course content is made attractive, which helps in minimizing the introversion and isolation of the students. The social constructive view of creating a community for a stronger bond between the learner and the trainer is to make new knowledge together that encourages their active participation and communication among them and creates such a stable environment where interacting with peers and instructors paves the way to learn new things from each other’s experiences (Shackelford and Maxwell, 2012). Therefore, support from the instructor and addressing the queries put make more frequent interaction with the instructor, which helps in creating a favorable environment that strengthens the bonds and contributes to environmental stability. It also maintains healthy relationships with each other by sharing ideas through e-learning. This helps in environmental sustainability by opting for the environmental awareness courses. On the basis of the literature above, the following hypotheses have been given.

*H* _10_
*: Mental health of students has an effect on their interaction with the instructor.*
*H* _11_
*: Interaction with the instructor has an effect on a sustainable environment.*
*H* _12_
*: Interaction with the instructor mediates the relationship between mental health and a sustainable environment.*

#### Community Support

Although the formation of a community that supports each other has its own difficulties associated with it, most of the communities are made to reinforce the support system of members in the social context (Coşkun and Kara, 2020). The community support among the students and other individuals works on the theory of social exchange (Nunkoo and Ramkissoon, 2011). According to this theory, the community and the people around you tend to help you only when they expect some social benefit in return at some other time in the future. However, the mental state of the people around us plays a very major role in shaping the behaviors of people (Liébana-Presa et al., 2020). Perceived benefits and costs are the major factors that make people behave in a certain way. If they are expecting a future benefit, they will be more satisfied by helping people around them. Therefore, it is important for the organizations and, especially, the educational institutes, in the case of the current study, that they take care of the students by making their support available to everyone involved in the class. Here lies the responsibility of the instructors to make such groups or teams in the class with diverse backgrounds so as to enrich the outcomes of the teams with different inputs, helping the low achievers in achieving high or polishing the skills of the introverts. Community support has been an important constituent of online learning for making online learning easy and not a burden to undergraduate students who are not mature enough to handle the stress of this new mode of learning alone. Therefore, the community support of online learners needs to be active and extend their help either to the family, friends, or colleagues to make them not feel depressed with the inability to understand and make the most out of it. Therefore, students starting the new semester and dealing with new content and technology in their course should be given an appropriate and supportive environment where the instructor supports and encourages the students. Thus, the following hypotheses are proposed in this regard:

*H* _13_
*: Mental health of students has an effect on community support.*
*H* _14_
*: Community support has an effect on a sustainable environment.*
*H* _15_
*: Community support mediates the relationship between mental health and a sustainable environment.*

#### Learning Management

The learning management in e-learning behavior is related to activities, such as planning and providing an effective learning atmosphere, which is necessary for the sustainability of the learning and creating a balanced learning environment (Raji and Zualkernan, 2016; Lee et al., 2019; Calvo et al., 2020; Nie et al., 2020). Learning management has certain benefits associated with it, such as the shift from content-based learning to process-based learning and helps in upgrading passive learning to active learning. However, this shift has been reported as painful for the concerned parties in some cases (Wang et al., 2012). When the students are able to properly manage the learning resources and organize the schedules for the learning activities makes it convenient for the students and the teachers. It has also been found that managing the learning activities by making the checklist or prioritizing the tasks makes the learning easier for the students. The management of the learning resources has changed the characterization of on-campus learning and has been found to have insightful effects on the learning activities of the students within universities. According to Wang et al. (2012), privacy and security on internet resources are the critical factors in the learning management of students. When the students tend to have sound and calm mental stability, they tend to manage and organize their learning materials and activities with more productive outcomes. Therefore, the next hypotheses are postulated as follows:

*H* _16_
*: Mental health of students has an effect on learning management.*
*H* _17_
*: Learning Management has an effect on a sustainable environment.*
*H* _18_
*: Learning management mediates the relationship between mental health and a sustainable environment.*

## Conceptual Framework

**FIGURE 1:**
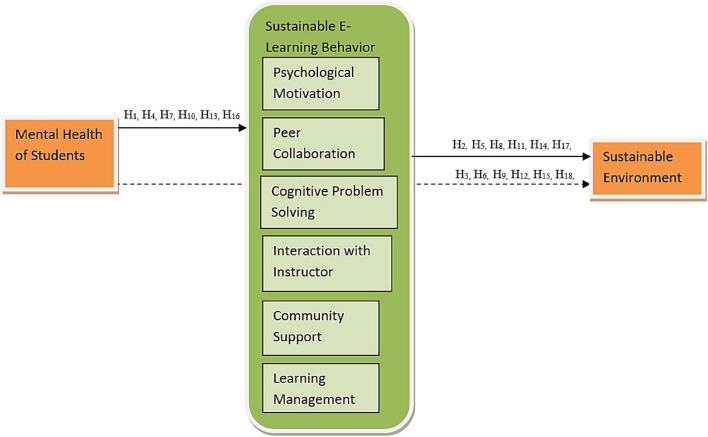
Theoretical framework. *N* = 340, CPS, cognitive problem-solving; CS, community support; IWI, interaction with instructor; LM, learning management; MH, mental health; PM, psychological motivation; PS, peer collaboration; SE, sustainable environment.

The following theoretical framework (see Figure 1) has been proposed in the study based on the gaps obtained from the literature. The current model operates on the goal theory, where individuals tend to develop a psychological and intrinsic motivation to attain their goals and the theory of sustainability which explains the achievement of optimum utilization of resources without compromising the availability of future resources (Ciocoiu, 2011). In previous studies, the mental health of students has been identified as a critical issue in the higher education sector, which is highly affected by the mental health of students. Previous studies considered that the on-campus environmental factors had been studied, while the online nature of the studies has yet to be studied (Devries et al., 2018). In this model, the change in the curriculum with online learning has been energized with a motive to obtain a sustainable environment with few environmental hazards and learn how to keep and maintain the goodness of the surroundings through certain collaborative social actions.

## Research Methods

The current study was conducted using the positivism approach to research. The positivism approach states that certain variables cause changes in other variables. Hence, this study measured the effect of mental health on sustainable e-learning behavior and its consequent role in the sustainable environment. Overall, this was a cross-sectional study that followed the quantitative techniques to analyze the data obtained from the surveys (Razak et al., 2019). Furthermore, it was a deductive frame of study as certain hypotheses have been formulated based on the support from prior literature, and different analytical techniques were used to measure the proposed relationships. The data collection was done by setting a questionnaire measuring each variable through the questions developed in the literature for measuring those particular variables. The data in the study were collected through a self-administered survey containing 37 items in total. The data sources used in this study were primary, and the sampling design used was convenience sampling as not all the potential respondents of the study were willing to fill the survey; hence, the convenience of the respondents was considered (Avotra et al., 2021a). The unit of analysis for the current study was undergraduate students in China (Peterson and Merunka, 2014). The students of the universities registered in the undergraduate programs were asked to fill the survey, addressing the variables of mental health, sustainable e-learning behaviors, and the sustainable environment. Therefore, the data were collected from the undergrad students in their current educational settings. The questionnaire was validated through different statistical tests usually used, i.e., HTMT ratio and Fornell and Larcker tests. The reliability of the scale was checked through Cronbach alpha and composite reliability. There were a total of 340 usable responses out of 400, which were used to run the analysis for structural equation modeling. The software used was Smart PLS 3.

### Instrument Measures

The questionnaire used in this study consisted of 37 items in total. The items were taken for each variable from the most accepted past pieces of research in the concerned field, making three main variables, i.e., mental health, sustainable e-learning behavior, and sustainable environment. The scale of sustainable e-learning behavior was made up of six sub-variables.

The scale of mental health, which is the independent variable of the study, was obtained from Zhou and Zhang (2021), which consisted of seven items. The sample items were: How often have you been bothered by the following feelings? (5 = not at all, 4 = several days, 3 = more than half of the days, 2 = nearly every day, 1 = two times or more every day) (1). Feeling nervous, anxious, or on edge, (2). Not being able to stop or control worrying (3). Being so restless that it is hard to sit still, (4). Becoming easily annoyed or irritable, (5). Feeling afraid as if something awful might happen, (6). Worrying too much about different things, and (7). Trouble relaxing.

The scale of sustainable e-learning consisted of 25 items in total, which was adapted from Lee et al. (2019) and was divided into six sub-variables. The psychological motivation consisted of 6 items; peer collaboration and cognitive problem-solving were made up of 5 items each; interaction with supervisors was made up of 2 items, while community support and learning management consisted of 3 and 4 items, respectively.

On the other hand, the dependent variable of the study, i.e., sustainable environment, consisted of 5 items, which were adapted from Biasutti and Frate (2017). The statements for each scale were adapted according to the needs of the study, and the scale of the study was developed on the five-point Likert scale, where 1 shows strong disagreement, while 5 shows strong agreement.

### Methodology

The data obtained in the study were analyzed using the well-known software Smart-PLS 3.3. Smart-PLS usually analyzes the data in two steps, i.e., measurement and structural. In the measurement model, the data are checked for preliminary validation and reliability so as to leave no loop in the results obtained for the measurement of the hypotheses in the structural model. The tests used for reliability are the Cronbach alpha reliability test and the composite reliability, while Heterotrait-monotrait tests are used along with the Fornell and Larcker test, checking the validity of the data (Avotra et al., 2021b). Furthermore, these tests of validity check the consistency of the constructs through convergent and discriminant validities. Average variances extracted (AVE) (0.5), factor loading (0.6), Cronbach alpha, and composite reliability should meet the threshold of 0.7 for acceptance (Avotra et al., 2021a).

### Data Analysis

The data in the study were analyzed in three stages. In the first stage, the demographic dynamics (see Table 1) of the respondents were checked, using age, gender, education, and the nature of their enrollment as hostellite or non-hostellite. The population was taken as undergraduates, while there were further categorized into three different classes addressing the under 12 years of education (high school), bachelors (currently enrolled), and others/diplomas (Peterson and Merunka, 2014).

**TABLE 1:** Demographics analysis.

Demographics	Frequency	Percentage
**Gender**		
Male	201	59.11%
Female	139	40.88%
**Age**		
15–20	103	30.29%
21–25	165	48.5%
26–30	72	21.17%
31 and above	-	-
**Education**		
12 years of education	172	50.58%
Bachelors	115	33.82%
Diploma and others	53	15.58%
**Nature of enrollment**		
Hostellite	192	56.47%
Non-hostellite	168	49.41%

*N = 340.*

**FIGURE 2:**
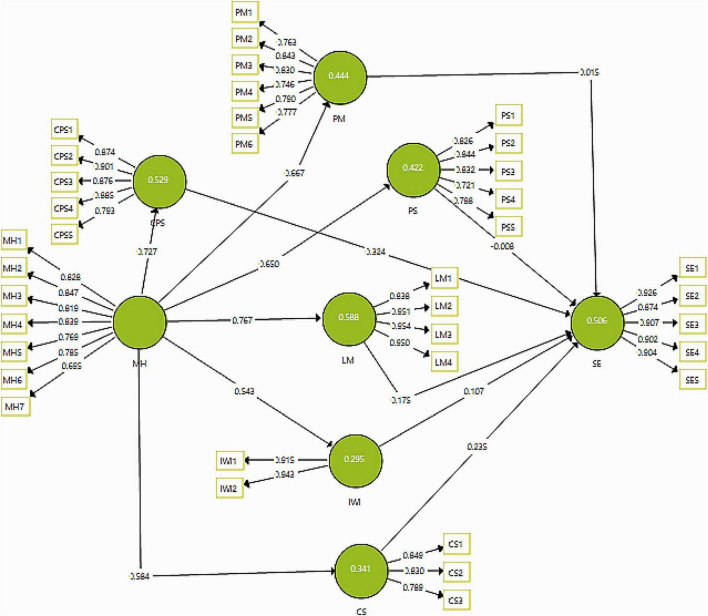
Measurement model analysis. *N* = 340, CPS, cognitive problem-solving; CS, community support; IWI, interaction with instructor; LM, learning management; MH, mental health; PM, psychological motivation; PS, peer collaboration; SE, sustainable environment.

In this study, the measurement model (see Figure 2) showed the construct reliabilities and the factor loadings of the indicators to show the significance of the indicators used in the study.

The results of the average variance-extracted factor loadings are shown in Table 2. All the values obtained in the factor loading from the measurement model showed all above 0.6 the threshold mentioned in the literature. Also, the coefficients of Cronbach alpha reliabilities and composite reliabilities for all the variables are well above 0.7, which is the minimum acceptable value (Amora, 2021). Similarly, the values for AVE are above 0.5, which is the cut-off value decided by the researchers (Hair et al., 2017; Franke and Sarstedt, 2019).

**TABLE 2:** Factor loadings and average variance extracted.

Variables	Factor loadings	Cronbach alpha	Composite reliability	AVE
Mental health	MH1 0.828	0.905	0.925	0.638
	MH2 0.847			
	MH3 0.819			
	MH4 0.839			
	MH5 0.769			
	MH6 0.785			
	MH7 0.695			
Psychological motivation	PM1 0.763	0.883	0.910	0.628
	PM2 0.843			
	PM3 0.830			
	PM4 0.746			
	PM5 0.790			
	PM6 0.777			
Peer collaboration	PS1 0.826	0.864	0.901	0.646
	PS2 0.844			
	PS3 0.832			
	PS4 0.721			
	PS5 0.788			
Cognitive problem solving	CPS1 0.874	0.916	0.938	0.751
	CPS2 0.901			
	CPS3 0.876			
	CPS4 0.885			
	CPS5 0.793			
Interaction with instructor	IWI1 0.915	0.842	0.926	0.862
	IWI2 0.943			
Community support	CS1 0.849	0.763	0.863	0.677
	CS2 0.830			
	CS3 0.789			
Learning management	LM1 0.838	0.942	0.959	0.854
	LM2 0.951			
	LM3 0.954			
	LM4 0.950			
Sustainable environment	SE1 0.838	0.943	0.957	0.815
	SE2 0.951			
	SE3 0.954			
	SE4 0.950			
	SE5 0.838			

*N = 340, AVE, average variance extracted.*

Furthermore, the validity measures of Fronell and Larcker and HTMT ration are mentioned in Table 3. The HTMT ratios were obtained for the study, which has the acceptance of the values if they are below 0.9. The values obtained in the table of HTMT ratio meet the criteria of Franke and Sarstedt (2019) of 0.9. Hence, the variables show discriminant validity.

**TABLE 3:** HTMT ratio.

	CPS	CS	IWI	LM	MH	PM	PS	SE
CPS								
CS	0.788							
IWI	0.646	0.648						
LM	0.634	0.619	0.493					
MH	0.792	0.687	0.610	0.827				
PM	0.529	0.658	0.558	0.614	0.719			
PS	0.541	0.425	0.370	0.507	0.710	0.781		
SE	0.697	0.715	0.559	0.577	0.623	0.484	0.389	

*N = 340, CPS, cognitive problem-solving; CS, community support; IWI, interaction with instructor; LM, learning management; MH, mental health; PM, psychological motivation; PS, peer collaboration; SE, sustainable environment.*

The values obtained in the Fornell and Larcker criterion have been found to be significant. The values in bold in the table are the highest of the rest of the values under it, hence showing the significance of discriminant validity for the measures of the current study. The results of the Fronell and Larcker test are reported in Table 4.

**TABLE 4:** Fronell and larcker.

	CPS	CS	IWI	LM	MH	PM	PS	SE
CPS	**0.867**							
CS	0.669	**0.823**						
IWI	0.569	0.534	**0.929**					
LM	0.591	0.533	0.447	**0.924**				
MH	0.727	0.584	0.543	0.767	**0.799**			
PM	0.497	0.563	0.525	0.590	0.667	**0.792**		
PS	0.499	0.360	0.333	0.481	0.650	0.681	**0.804**	
SE	0.649	0.608	0.500	0.545	0.577	0.463	0.369	**0.903**

N = 340, CPS, cognitive problem-solving; CS, community support; IWI, interaction with instructor; LM, learning management; MH, mental health; PM, psychological motivation; PS, peer collaboration; SE, sustainable environment. The values in bold in the table are the highest of the rest of the values under it, hence showing the significance of discriminant validity for the measures of the current study.

The r square and f square values have been reported in Table 5. The highest r-square has been observed for learning management (r-square = 0.588) followed by cognitive problem-solving (r-square = 0.529) followed by psychological motivation (r-square = 0.444), and peer collaboration (r-square 0.422). A statistical value for F-square less than 0.02 shows a small effect size, a value is less than 0.15 shows moderate effects, and values greater than 0.35 show strong effects. Similarly, the highest values for f-square are seen for learning management (f-square = 1.426) and cognitive problem-solving (f-square = 1.124), which are very good indicators of the significance of the variable. The rest of the values for r-square and f-square can be seen in Table 5.

**TABLE 5:** R-square and F-square.

Variables	R-square	F-square
Psychological motivation	0.444	0.799
Peer collaboration	0.422	0.730
Cognitive problem solving	0.529	1.124
Interaction with instructor	0.295	0.419
Community support	0.341	0.517
Learning management	0.588	1.426

**FIGURE 3:**
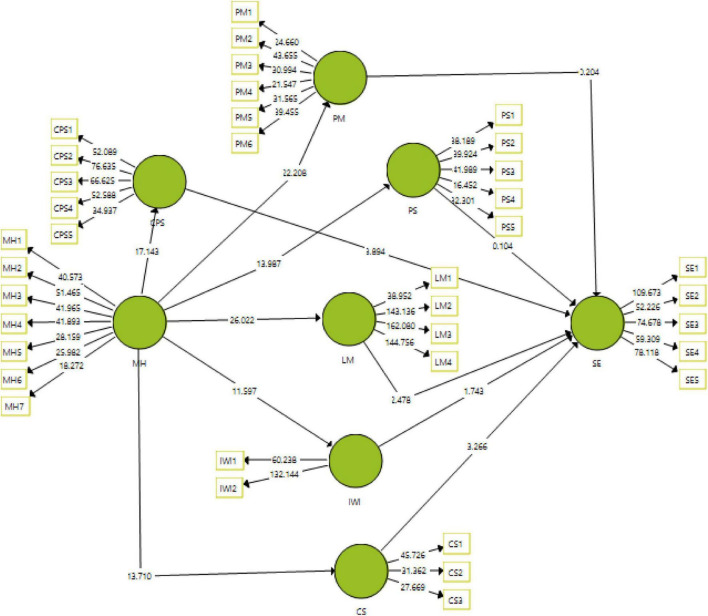
Structural model measurement.

Hypotheses of the study are measured with the help of structural modal estimations that show the significance of the results, thus helping in accepting and rejecting the hypotheses according to the preliminary set criterion. This study uses t-statistics, *p*-values, R-square, F-square, and original sample means to accept or reject the hypothesis. The level of significance used in this study was 0.05. The graphical representation of the structural model is given in Figure 3.

The results obtained from the structural model of measurement have been reported in Table 6. The first hypothesis for the association of mental health is found significant with t-statistic = 22.208 and *p* < 0.005. Similarly, the Hypotheses H_4_, H_7_, H_10_, H_13_, H_14_, H_16_, and H_17_ have been found significant in the study, having H_4_, i.e., an association of mental health with peer collaboration (t-statistic = 13.987), H_7_: association of mental health with a cognitive problem-solving (t-statistic 17.143) for H_10_: association of mental health with interaction with the instructor (t-statistic = 11.597) for H_13_: association of mental health with community support (t-statistic = 13.710) and for learning management t-statistic = 26.022 were all found significant at *p* < 0.001, thus accepting the respective hypotheses. However, psychological motivation, peer collaboration, and interaction with the instructor could not find significant results in a sustainable environment. The results have been reported in Table 6. Moving to the hypotheses H_8_: association of cognitive problem-solving with the sustainable environment (t-statistic = 3.894), H_14_: association of community support with the sustainable environment (t-statistic = 3.266), and H_18_: association of learning management with the sustainable environment (t-statistic = 2.478), they have been found significant at *p* < 0.05, hence accepting these hypotheses.

**TABLE 6:** Direct effects.

Paths	H	O	M	SD	T-statistic	*P*-value	Results
MH → PM	H_1_	0.667	0.666	0.030	22.208	0.000***	*Accepted*
PM → SE	H_2_	0.015	0.014	0.076	0.204	0.839	Rejected
MH → PS	H_4_	0.650	0.647	0.046	13.987	0.000***	*Accepted*
PS → SE	H_5_	−0.008	−0.004	0.073	0.104	0.917	Rejected
MH → CPS	H_7_	0.727	0.728	0.042	17.143	0.000***	*Accepted*
CPS → SE	H_8_	0.324	0.319	0.083	3.894	0.000***	*Accepted*
MH → IWI	H_10_	0.543	0.547	0.047	11.597	0.000***	*Accepted*
IWI → SE	H_11_	0.107	0.107	0.061	1.743	0.082	Rejected
MH → CS	H_13_	0.584	0.590	0.043	13.710	0.000***	*Accepted*
CS → SE	H_14_	0.235	0.236	0.072	3.266	0.001*	*Accepted*
MH → LM	H_16_	0.767	0.767	0.029	26.022	0.000***	*Accepted*
LM → SE	H_17_	0.175	0.178	0.071	2.478	0.014*	*Accepted*

N = 340, ***p < 0.001, **p < 0.005, *p < 0.05, H, hypothesis; O, original sample; M, sample mean; SD, standard deviation; CPS, cognitive problem-solving; CS, community support; IWI, interaction with instructor; LM, learning management; MH, mental health; PM, psychological motivation; PS, peer collaboration; SE, sustainable environment.

Furthermore, the indirect effects of the variables have also been studied, as depicted in Table 7. Among these relationships, cognitive problem-solving, community support, and learning management have been found significant, accepting H_9_: mediation of cognitive problem-solving in the relationship of mental health and sustainable environment (t-statistic = 3.666), H_15_: mediation of community support in the relationship of mental health and sustainable environment (t-statistic = 3.157), and H_18_: mediation of learning management in the relationship of mental health and sustainable environment (t-statistic = 2.549), which have been found significant.

**TABLE 7:** Direct effects.

Paths	H	O	M	SD	T-statistic	*P*-value	Results
MH → PM → SE	H_3_	0.010	0.009	0.050	0.204	0.839	Rejected
MH → PS → SE	H_6_	−0.005	−0.002	0.048	0.103	0.918	Rejected
MH → CPS → SE	H_9_	0.236	0.233	0.064	3.666	0.000***	*Accepted*
MH → IWI → SE	H_12_	0.058	0.059	0.035	1.664	0.097	Rejected
MHCS → SE	H_15_	0.137	0.139	0.043	3.157	0.002***	*Accepted*
MHLM → SE	H_18_	0.134	0.136	0.055	2.459	0.014*	*Accepted*

N = 340, ***p < 0.001, *p < 0.05, H, hypothesis; O, original sample; M, sample mean; SD, standard deviation, CPS, cognitive problem-solving; CS, community support; IWI, interaction with instructor; LM, learning management; MH, mental health; PM, psychological motivation; PS, peer collaboration; SE, sustainable environment.

## Discussion

Research on the mental health of the people during this pandemic, in particular, has been the center of attention for many researchers (Knowles, 2001; Lim et al., 2008; de Silva and Roland, 2014; Ohrnberger et al., 2017; Dybdahl and Lien, 2018; Groth et al., 2019; Filipova et al., 2020; Horesh et al., 2020; Lee, 2020; Liébana-Presa et al., 2020; Saricali et al., 2020; Adom et al., 2021; Bolatov et al., 2021; Chaturvedi et al., 2021; Deshpande and Mhatre, 2021; Gianfredi et al., 2021; Lister et al., 2021; Saha et al., 2021; Xiong et al., 2021). However, this has taken a new turn with the changing paradigm of learning behaviors from classroom face-to-face education to electronic learning of the students (Moore et al., 2011; Lahti et al., 2014; Cidral et al., 2018; Hasan and Bao, 2020; Lasfeto, 2020; Mousavi et al., 2020; Nie et al., 2020; Thandevaraj et al., 2021). The current study has been designed to answer the most prevailing interrogations of this post-pandemic scenario. Hence, the study was objectified by finding the answers to a few questions. The first goal of the study was to understand the role of mental health in the online learning behavior of undergraduate students who were not very mature to tackle the stress and anxiety of loneliness and social distancing in this pandemic. Second, to identify the effects of e-learning behavior on achieving a sustainable environment and third, to know how much does e-learning mediates the relationship between mental health and a sustainable environment. In order to answer these questions, this quantitative study was conducted in China.

This cross-sectional study took the population frame of undergraduate students enrolled in China, and their studies were interrupted due to the pandemic, and their modes of learning changed from conventional classroom learning to online learning. Therefore, it was focused on learning the mental condition of the students concerning their e-learning behavior in terms of psychological motivation, peer collaboration, cognitive problem-solving, interaction with instructors, community support, and learning management and how these factors can contribute toward the achievement of a sustainable environment. The data collected had been validated earlier with the factor loading of the indicators, Cronbach alpha coefficients, and the composite reliabilities. The values obtained from the data collected in the study were all rightly according to the threshold mentioned in the literature for factor loading, alpha, and composite reliabilities, HTMT ratio, and Fornell and Larcker criterion.

In this study, mental health has found a significantly stronger relationship with sustainable e-learning behavior that shows that the mental health of the students plays a very important role in the sustainability of their e-learning behaviors. The results suggested that among all the sub-factors of the e-learning behavior proposed by Lee et al. (2019), they have been the most considered perspectives of online learning that are affected by their mental health. The findings of the study on the role of mental health in e-learning behavior are due to the fact that when individuals are not in the right state of mind, they tend to see negativity from even the positive things that ruin their mood and hence the consequent behavior, where online learning is one of them due to the pandemic (Bolatov et al., 2021). The findings of the study strengthen the findings of the previous studies that have also found a role of mental health in the achievement of students and also their social behaviors with their peers and down of their psychological morale (Cao et al., 2020; Bolatov et al., 2021; Lister et al., 2021; Saha et al., 2021; Smith et al., 2021; Xiong et al., 2021; Zhou and Zhang, 2021).

Similarly, regarding the sustainability of the environment as a result of online learning, the current study has found that sustainable development goals are employed in higher education, which can be used in shaping sustainable behaviors by exercising education for a sustainable environment. These findings are due to the fact that awareness and extensive research are playing a very important role in helping educational policy makers reshape their curriculum according to the new demands of the growing and well-aware world. The awareness for a sustainable environment has been the key factor in the universities and colleges while delivering the content of the studies (Godemann et al., 2014; Sivapalan et al., 2016; Mahat et al., 2017; Badea et al., 2020). The findings of the study are in accordance with the previous studies conducted by many authors. They also found a relationship between the students’ behaviors and educational sustainability (Badea et al., 2020; Nie et al., 2020).

## Conclusion

The mental health of students has previously been found to have an impact not only on their daily lives but also on their study patterns and motivations. The present study has extended the literature by exploring the role of mental health of students in their educational studies, especially e-learning behavior due to the pandemic, which has shifted classroom learning to the online learning mode. It has been found that the mental health of the students has been a major predictor of the sustainable e-learning behavior of the students. It has also been found that if the students are mentally stable, then they can be more prone to making the environment sustainable with their cognitive and problem-solving skills by indulging in those activities, which cause less hazard to the environment.

## Practical Implications

The current study will help educational institutions improve their e-learning programs by understanding the preferences and challenges of the students regarding online learning. Educational institutes should make policies regarding online education and teaching methodologies that make learning easier for students so as to avoid the factor of mental health. This would help the higher education institutes in coping with any such disaster or emergency circumstances that demand remote online learning of the students. Furthermore, the government should make all efforts in making the materials and equipment necessary for making online learning available to all those who need it. The organizations can pool a social welfare fund to help the needy learners by delivering such programs that help the students with technical assistance. The institutes can make the strategies according to the learning preferences of the students and the teachers’ teaching methodologies to enhance their productive outcomes.

## Limitations of the Study

However, there are certain limitations as well, associated with the study. Future attempts can be made to measure these variables by taking time-series data, checking the post-pandemic and pandemic mental health of students. The current study has taken the undergraduate students as the sample. In future studies, these relationships can be checked in higher education as well. Furthermore, the framework of the study can be improved by taking more relevant variables and checking their moderation in these relationships, such as other work-from-home setups, considering the variables as team learning, moderating roles of task interdependence, personal competition, etc.

## Data Availability Statement

The original contributions presented in the study are included in the article/supplementary material, further inquiries can be directed to the corresponding author/s.

## Ethics Statement

The studies involving human participants were reviewed and approved by the Chengdu University of Technology, China. The patients/participants provided their written informed consent to participate in this study. The study was conducted in accordance with the Declaration of Helsinki.

## Author Contributions

ZG and PL conceived, designed, and wrote the manuscript. AZ and XX helped in data collection. FG helped with resources. All authors read and agreed to the published version of the manuscript.

## Conflict of Interest

The authors declare that the research was conducted in the absence of any commercial or financial relationships that could be construed as a potential conflict of interest.

## Publisher’s Note

All claims expressed in this article are solely those of the authors and do not necessarily represent those of their affiliated organizations, or those of the publisher, the editors and the reviewers. Any product that may be evaluated in this article, or claim that may be made by its manufacturer, is not guaranteed or endorsed by the publisher.
